# High molecular mass proteomics analyses of left ventricle from rats subjected to differential swimming training

**DOI:** 10.1186/1472-6793-12-11

**Published:** 2012-09-05

**Authors:** Luiz A O Rocha, Bernardo A Petriz, David H Borges, Ricardo J Oliveira, Rosangela V de Andrade, Gilberto B Domont, Rinaldo W Pereira, Octávio L Franco

**Affiliations:** 1 Centro de Análises Proteômicas e Bioquímicas, Programa de Pós-Graduação em Ciências Genômicas e Biotecnologia, Universidade Católica de Brasília, Brasília-DF, Brazil; 2 Programa de Pós-Graduação em Educação Física, da Universidade de Brasília; 3 Departamento de Biologia, Universidade Federal de Juiz de Fora, Juiz de Fora-MG, Brazil; 4 Universidade Federal do Rio de Janeiro, Protemics Unit, Rio de Janeiro, Brazil; 5 Pos-graduaÃ§Ã£o em patologia molecular, Universidade de Brasilia, Brasilia, DF, Brazil

**Keywords:** Heart tissue, High molecular mass proteomic, Muscle, Myofibrillar proteins, Swimming training

## Abstract

**Background:**

Regular exercises are commonly described as an important factor in health improvement, being directly related to contractile force development in cardiac cells.

In order to evaluate the links between swimming exercise intensity and cardiac adaptation by using high molecular mass proteomics, isogenic Wistar rats were divided into four groups: one control (CG) and three training groups (TG’s), with low, moderate and high intensity of exercises.

In order to evaluate the links between swimming exercise intensity and cardiac adaptation by using high molecular mass proteomics, isogenic Wistar rats were divided into four groups: one control (CG) and three training groups (TG’s), with low, moderate and high intensity of exercises.

**Results:**

Findings here reported demonstrated clear morphologic alterations, significant cellular injury and increased energy supplies at high exercise intensities. α-MyHC, as well proteins associated with mitochondrial oxidative metabolism were shown to be improved. α-MyHC expression increase 1.2 fold in high intensity training group when compared with control group. α-MyHC was also evaluated by real-time PCR showing a clear expression correlation with protein synthesis data increase in 8.48 fold in high intensity training group. Other myofibrillar protein, troponin , appear only in high intensity group, corroborating the cellular injury data. High molecular masses proteins such as MRS2 and NADH dehydrogenase, involved in metabolic pathways also demonstrate increase expression, respectily 1.5 and 1.3 fold, in response to high intensity exercise.

**Conclusions:**

High intensity exercise demonstrated an increase expression in some high molecular masses myofibrilar proteins, α-MyHC and troponin. Furthermore this intensity also lead a significant increase of other high molecular masses proteins such as MRS2 and NADH dehydrogenase in comparison to low and moderate intensities. However, high intensity exercise also represented a significant degree of cellular injury, when compared with the individuals submitted to low and moderate intensities.

## Background

The adaptation is a dynamic process with involvement of many circumstances and is an important life mechanism. The cardiac cells of mammals are submitted to a growth phase after birth maturation period defied as cardiac hypertrophy which is characterized by n increase in individual size of the cardiomyocyties without cell division. This pattern of hypertrophy development can be initiated in response to some intrinsic and extrinsic stimuli such mechanical stress, neurohumoral factor, cytokines, ischemia and endocrine disorders [1,2].

These stimuli for heart hypertrophy can be divided in “physiological” cardiac hypertrophy, when is result of exercise for example and “pathological” cardiac hypertrophy, which is associated with cardiovascular diseases [1,2]. The physiological hypertrophy has been classified as a positive increase in heart mass, associated with structural remodeling of components of the ventricular walls to support increase in myocyte size, angiogenesis and changes in fibrilar collagen content and organization, whose enhancer the cardiac pump function.

The cellular and molecular bases behind heart adaptations to exercise are not completely understood, but it is believed that a number of cellular adaptations, intrinsic to the cardiomyocyte, are largely responsible for these changes. The mechanism proposed for this structural adaptation is hypertrophy by increase in functional load [3]. This overload is followed by modifications in the gene expression pattern, activation of signaling pathways which included up regulation in the contractile protein synthesis and his organization into sarcomeric units [1-3]. However, it is not clear what role is played by the impact of differing training intensities on the physiological heart muscle’s adaptation, in terms of molecular changes.

In summary the literature describes that one of the most important benefits of exercise, associated to cardio-circulatory system consists in the enhancing of heart work efficiency due to contractile capacity increase. Thus, this study aims to verify the rat cardiac muscle adaptation under different intensities of swimming exercise, focusing on high molecular mass proteomics of *R. novergicus.*

## Results and discussion

Measured average values showed a lower lactate accumulation, of 4.6 (± 0.7 mmol.L^-1^ in TG_3_ in comparison to 5.9 ± 0.4 mmol.L^-1^ in CG). The two other TG’s (data not shown) also showed lower value, [4]. Cardiac fibers from left ventricle of rats in the CG showed a normal size and shape (Additional file 1: Figure S1). Similar data were obtained from left ventricle histological sectioning of rats from TG_1_ (Additional file 1: Figure S1), demonstrating no structural alteration in the heart tissue, which indicated lower hypertrophy. Exercise training was also able to induce heart morphological alterations not seen in the untrained group. The small nucleus presented a rounded or in some cases slightly oval shape, indicating no clear pathological alteration which lower hypertrophy. A few morphologic alterations, such as a small sclerotic area with infiltration of granule lipofuscin, characterizing particular metabolic alterations, probably associated with overload were seen in TG_2_, (Additional file 1: Figure S1) [5,6]. This pigment is closely associated with oxygen-derived free radicals, which are an important component of muscle fatigue [7] indicating that TG_2_ are inducing heart tissue modifications to the detriment of improvement in metabolism. On the other hand, microscopic morphology analysis in TG_3_ indicated cellular hypertrophy, showing several modifications such as several areas with increased fibrosis (Additional file 1: Figure S1), evidencing a higher adaptation to exercise overload. This increase suggests that this level of physical activity was stressful to the heart [8,9]. The relative exercise overload is directly associated with oxygen consumption (VO_2máx_) [10,11], because mitochondria are strictly involved in the activation of super-oxide synthesis cascade. It is also worth noting that TG_3_ showed an extended area with necrosis and the presence of leucocytes, probably supporting hypertrophy by replacing dead cells with satellite cells (Additional file 1: Figure S1) [12,13]. One important route to hypertrophy of the myocardium directly involves fibroblast proliferation, which stimulates collagen synthesis [14-16]. Increased collagen content is commonly observed with overload pressure and, in certain cases, may negatively impact both diastolic and systolic function [17]. One of the most important benefits of exercise in the cardio-circulatory system is associated with an increase in circulation capability. This upgrade is strictly dependent on contraction increase, carried out by the expression of different heart myosin isoforms [18-22], which will be described below.

### Differential electrophoretic analyses of sedentary and exercise-trained cardiac muscle

**Figure 1:**
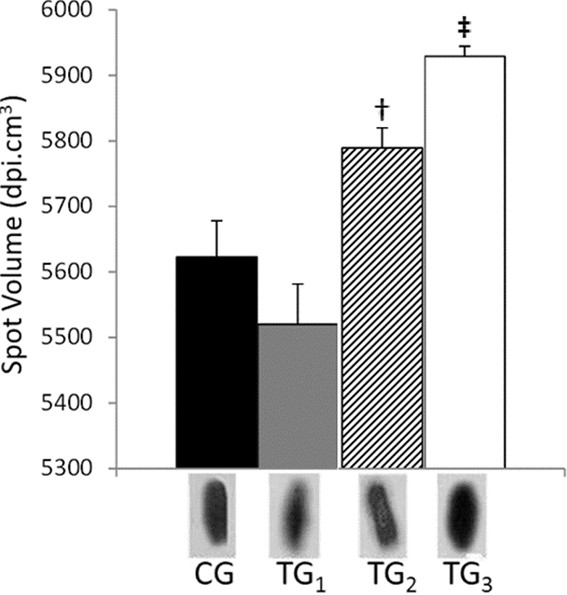
**α-MyHC protein quantity analyses.** The α-MyHC expression was evaluated by high molecular mass 2-DE technique in rat cardiomyocytes of the left ventricle. CG corresponds to control group; TG_1_; TG_2_ and TG_3_ correspond respectively to 2.5; 5.0 and 7.5 to training groups. Different spots volumes are determinate by using software Bionumerics™. Symbols † and **‡** represent the statistical difference between respectively TG_2_ and TG_3_. Statistical analyses were performed by ANOVA ( P < 0.05). All studies were performed in triplicate.

Since exercise-trained groups showed clear heart tissue alterations, SDS-PAGE was conducted in order to evaluate protein expression modification, showing an apparent quantitative increase in α-MyHC (higher band) and β-MyHC (lower band) expression (Additional file 2: Figure S2), in TG_2_ and TG_3_ compared to CG. Analyzing overlapped images, it was observed that α-MyHC (Figure 1) increased 1 fold in swimming-trained heart. In order to improve these data, increasing accuracy and leading to an overview of physiologic modifications during swimming exercise training, two-dimensional gels were run by using left ventricles from control and training groups (Additional file 3: Figure S3). Bionumerics™ from Applied Maths matched 177 spots in the 2-DE control group.

It is known that two MyHC isoforms, α-MyHC and β-MyHC, are expressed in cardiac tissue, and their proportion (normally ~ 70% α-MyHC and 30% β-MyHC) in the rodent myocardium directly influences heart power output [23,24]. Data reported here are consistent with previous results in which MyHC was up regulated in exercise-trained rat hearts [11]. Otherwise, these data contradict those observed during moderate exercise training on skeletal rat muscle suffering chronic heart failure [25]. In this case, MyHC distributions were similar in both groups and training did not alter the MyHC distribution.

We found 162 spots in TG_1_, 168 in TG_2_ and 186 in TG_3_. The number of spots observed in each gel (Table 1), was lower than previously observed in rat heart protein maps (624 spots) submitted to intensity-controlled endurance exercise [11]. This variation could be explained by a different 2-DE technique here utilized, which is focused on evaluation of high molecular mass proteins ranging from 50–220 kDa, while Burninston [11] evaluated most abundant proteins from 14 – 116 kDa. The average coefficient of variation for normalized spot volume gave R^2^ = 0.82 for biological and 0.94 for technical replicates, showing reliable gel reproducibility. These values are close to the range reported for technical variation for proteomic analyses of muscle homogenate, including skeletal and heart muscle tissues [4,11,26]. Moreover, the average coefficient of variation calculated to compare gels from different groups (CG, TG_1_, TG_2_ and TG_3_) showed a R^2^ lower than 0.43, demonstrating the obvious differences in protein maps from rat left ventricles submitted to diverse intensities of training. These proportions were similar to that observed by Burniston [11], evaluating the adaptation of rat cardiac muscle to endurance exercise, despite the different methodologies utilized. While Burniston described the evaluation of proteins from 14 to 116 kDa [27], Our work evaluated proteins varying from 53 to 230 kDa, indicating that protein expression behavior in response of exercise is independent of molecular masses. Moreover, these data are only in agreement with protein maps from rat skeletal muscle [4] and human heart [28]. Furthermore, as previously observed in several reports [4,11], some gene products were identified as multi-spot series exhibiting similar molecular masses but different pI, which may indicate unusual splice variants or states of post-translational modification. 

**Table 1:** MALDI ToF protein identification

**Samples**	**α-MyHC**	**18srRNA**	Δ**C**_**T**_	ΔΔ**C**_**T**_	**α-MyHC expression**
	**average**	**average**			**increase in TGs fold related to CG**
	**C**_**T**_	**C**_**T**_			
Control	26.31 ± 0.12	15.21 ± 0.55	11.10 ± 0.32	0.00 ± 0.32	1.00 (0.80-1.25)
TG_1_	25.58 ± 0.10	15.16 ± 0.46	10.42 ± 0.27	-0.68 ± 0.27	1.60 (1.33-1.94)
TG_2_	25.32 ± 0.06	15.39 ± 0.56	9.92 ± 0.32	-1.18 ± 0.32	2.26 (1.81-2.83)
TG_3_	26.28 ± 0.04	18.26 ± 0.63	8.02 ± 0.36	-3.08 ± 0.36	8.48 (6.59-10.91)

Real time PCR evaluating fold change expression of α-MyHC RNA from training groups (TGs) related to untrained group (CG) calculated by ΔΔCT method. Values were presented as a means ± standard deviation.

### Mass spectrometry protein identification

Of all spots founded seventeen had been considered differentially expressed after swimming training, whereas increase more than one fold and appear in every gel in triplicate. Those were identified by PMF (Table 1) with molecular masses above 56 kDa. These data revealed that the majority (27%) of identified proteins were mitochondrial, one-quarter associated with membrane/ extracellular (25%), one-quarter myofibrillar/cytoskeletal (25%) and 23% associated with different functions such as hystocompatibility (Table 1). Besides the evidence, based on protein analysis, of increased expression of α-MyHC in our training groups, we decided to corroborate it using mRNA expression (Table 2). Data here reported showed that training intensity is an important factor for α-MyHC expression. Training groups with overload of 2.5 (TG_1_) and 5% (TG_2_) showed increased expression of α-MyHC related to untrained group (CG). Respectively, these were increases of 1.60 and 2.26 fold in α-MyHC expression. However, taking in account standard deviation, the increase in TG_1_ was 1.33-1.94 and in TG_2_ it was 1.81-2.83-fold. The overlap in the upper limit of TG_1_ with lower limit in TG_2_ shows lower differentiation in these two training groups for α-MyHC expression. The training group with overload of 7.5% (TG_3_) showed an 8.48-fold (6.59-10.91) increase.

**Table 2:** Real time PCR results

**Spot**	**Obs. pI**	**Exp. pI**	**Obs. M.M.(kDa)**	**Exp. M.M.(kDa)**	**Sequ. Match.**	**Sequ. not match.**	**Cover.%**	**e- value**	**Swissprot code**	**Protein**	**Protein funtion**	**CG**	**TG1**		**TG2**		**TG3**	
												**Regul. factor**	**Regul. factor**	**p-value**	**Regul. factor**	**p-value**	**Regul factor**	**p-value**
1	9.0	9.4	≈ 120	122	25	84	23.3	0.4	Q8K4V5	High-affinity immunoglobulin gamma Fc receptor I.	Phagocytes process	1.00	absent	-	absent	-	1.01	0.05
2	8.1	9.2	248	34.4	70	239	22.7	0.3	Q3KRD5	Mitochondrial import receptor subunit TOM34.	Translocase of outer membrane 34 kDa subunit associated to energy synthesis.	1.00	absent	-	absent	-	1.32	0.05
3	6.9	5.9	175	105	33	55	37.	0.1	Q60I07	MHC class I-like located near the LRC, 1.	Immunological leukocyte response.	1.00	absent	-	absent	-	1.00	0.01
4	4.3	9.7	≈ 250	227	65	135	32.5	1.0	AAH83554	Magnesium transporter MRS2.	Mg^2+^mitochondria’s transporter	1.00	1.12	0.05	1.27	0.05	1.53	0.01
5	5.9	5.2	≈ 230	364	63	276	18.6	0.1	Q924V0	Cadherin class 1 receptor.	Immune system.	1.00	absent	-	absent	-	absent	-
6	5.2	5.7	≈ 100	314	72	213	25.3	0.6	BAA18993	N-G,N-G-dimethylarginine dimethylaminohydrolase.	NO synthetases inhibitor.	1.00	absent	-	1.00	0.05	1.01	0.05
7	6.4	9.1	120	127	630	477	56.9	0.08	Q63356.1	α-Myosin heavy chain.	Primary motor of muscle contraction on cardiac cells.	1.00	1.04	0.05	0.93	0.01	1.24	0.05
8	5.5	9.2	193.9	34.4	54	185	22.6	0.4	Q3KRD5	Mitochondrial import receptor subunit TOM34.	Translocase of outer membrane 34 kDa subunit associated to energy synthesis.	1.00	absent	-	1.47	0.05	1.36	0.05
9	6.9	5.2	192.6	107	698	277	71.6	0.04	AAF37622	Glutamyl aminopeptidase.	Rennin-angiotensin catabolism pathway.	1.00	1.30	0.05	1.10	0.05	1.32	0.05
10	7.3	9.8	213.0	82	48	270	15.1	0.09	CAA40164	NADH dehydrogenase, mitochondrial subunit 1.	Catalyzes the first dehydrogenase reaction in the TCA cycle	1.00	1.07	0.05	1.18	0.05	1.50	0.05
11	5.8	9.4	223.1	46	52	36	59.1	0.1	Q60I18	Major histocompatibility complex class 1.	Immunological leukocyte response.	1.00	absent	-	absent	-	1.12	0.05
12	8.3	9.6	224.9	215	76	135	36.0	1.0	P23693	Troponin I, cardiac muscle**.**	Muscular contraction regulator by its relation with calcium molecules.	1.00	1.19	0.01	1.05	0.05	1.26	0.001
13	5.6	9.2	240.0	34.4	58	185	23.9	1.0	Q3KRD5	Mitochondrial import receptor subunit TOM34.	Associated to energy production.	1.00	absent	-	absent	-	1.42	0.08
14	4.8	9.6	232.2	21	44	167	20.9	2.2	P23693	Troponin I, cardiac muscle**.**	Muscular contraction regulator by its relation with calcium molecules.	1.00	1.00	0.05	1.00	0.05	1.1	0.05
15	5.5	6.1	249.9	42	26	62	29.5	1.0	Q861Q1	Major histocompatibilitycomplex class 1	Involved in the immunological leukocyte response.	1.00	1.00	0.05	1.02	0.05	1.31	0.05
16	8.6	9.2	261	34.4	73	236	23.6	0.8	Q3KRD5	Mitochondrial import receptor subunit TOM34.	Energy synthesis	1.00	absent	-	absent	-	1.0	0.05
17	4.7	5.2	225	107	66	909	6.8	0.04	AAF37622	Glutamyl aminopeptidase.	Rennin-angiotensin catabolism pathway	1.00	absent	-	1.03	0.05	1.13	0.05

Differential proteins identified by MALDI ToF peptide mass fingerprinting of multiple proteins from rat left ventricles. As a standard, false Discovery rate was calculated as lower than 15%.

**Figure 2:**
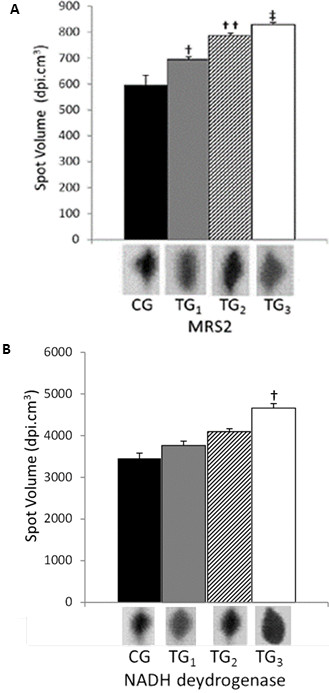
**MRS2 and NADH protein quantity analyses.** MRS2 (**A**) and NADH dehydrogenase (**B**) expression evaluated by high molecular mass 2-DE technique in rats left ventricle cardiomiocities. CG corresponds to control group; TG_1_; TG_2_ and TG_3_ correspond respectively to 2.5; 5.0 and 7.5 to training groups. Different spots volumes are determinate by using software Bionumerics™. Simbols **†**, **††** and **‡**, represent the statistical difference between respectively groups. Statistical analyses were conducted by ANOVA (P < 0.05). All studies were performed in triplicate.

Aiming to unambiguously differentiate α- and β-MyHC, one ion, which is unique to α-MyHC, of 2036.48 m/z corresponding to residues 1546–1663 (KNAQAHLKDTQLQLDDAVRA) was sequenced from spot 7. Tryptic peptides specifically from β -MyHC were not detected, suggesting that spot 7 is formed by α-MyHC. A similar strategy to elucidate MyHC isoforms has been used before [11]. This result clearly showed a significant increase in α-MyHC expression in proportion to training intensity. A clear cut among TG_3_ and the other two training groups was also seen in histopathological analysis and electrophoretic analyses, which showed that TG_3_ was clearly able to improve α-MyHC expression (Figure 2). Since α-MyHC up regulation in exercised-trained rat hearts is consistent with previously published data [11,29,30], here we could conclude that intensity, at least in swimming training, is the key to controlling α-MyHC expression and indeed cardiomyocite power output (Figure 1). Other studies using swimming as a training method have suggested that this practice could induce an increase in α-MyHC expression in the rat heart [19]. The same author described little modifications in MyHC isoform content in trained myocardium, despite finding increase in loaded shortening velocity. These results suggest that it is possible to have significant alterations in myocardial contractile function with lower or no change in MyHC isoform expression, as was observed here. However, despite changes in the myosin subunit isoform expression are associated to increase in contractility by an increase in force generation, economy in ATP ratio and increased Ca^2+^ sensitivity, others low molecular weight sarcomeric protein, that they had not been described in this work, like myosin regulatory light chain, troponin-I, troponin-T, alpha-tropomyosin and myosin binding protein-C must be considered for yours relevance for contractile mechanism [31]. 

Since the 1980s some important studies have reported that an increase in α-MyHC from exercise [32-34] is able to boost cardiomyocyte power output [4,11], especially for myofibrillar proteins [4]. Here, in this report, we used a combination of protein extraction under high salt quantities associated with a long and modified 2D gel run with low acrylamide concentration (8%). These procedures allowed the visualization of high molecular mass protein maps. Otherwise, spot resolution was lower when compared to other muscle tissues’ protein maps [4,11]. Similar data were obtained with treadmill-trained rats, in which a 1.9-fold increase of α-MyCH expression was observed [11]. Spot 7 showed 120 kDa of molecular weight, which is approximately half that of the predicted myosin heavy chain complete polypeptide. Furthermore, several MyHC isoforms (17) could share 93% sequence identity [11].

Troponin, which acts as a muscular contraction regulator due to its relationship with calcium molecules, was identified in two different spots (Table 1). It is important to note that, probably, a different troponin species, which showed identical molecular mass (approximately 220 kDa), presented a completely different pI. While one species extracted from spot 14 showed a pI of 4.8, the troponin species from spot 12 had a pI of 9.6 (Table 1). The troponin from spot 12 was only observed in protein maps from rat hearts submitted to intense exercise (TG3), suggesting that only intense exercise is capable of improving its expression at 2-DE detectable levels (data not shown).

Troponin is commonly utilized as a serum muscle injury marker in cardiac insufficiency and, consequently, pathologic hypertrophy [27,35,36]. For Lippi and Banfi, an increase in cTns values might be temporarily responsible for a reversible shed of cardiac blebs. Elevated levels of cTn could be due to ongoing myocardial damage or leakage of myofibrillar components and may reflect the loss of viable cardiac myocytes [37]. Myocyte injury, coronary microvascular dysfunction, and fibroblast and collagen turnover also play an important role in cardiac remodeling; extracellular matrix remodeling takes place in myocardium hypertrophy [38]. Nie et al. [39] suggest a role for exercise-induced increases in ROS in the mediation of cTn. Moreover, oxidative stress in high exercise intensity induced microvascular flow abnormalities, which in turn are associated with an increase in cTn levels observed by microscopic analyses [40]. To support this fact, the presence of an isoform of major histocompatibility complexes, that are strictly associated with immune response in nucleated cells, such as necrosis or hydrolases enzymes, was detected at higher levels in TG_3_. This process distinguishes between self-proteins and foreign protein antigens to elicit an effective immune response called antigen-presenting cells [41]. These data, in addition to histological analysis previously described, which show a large necrosis area as well as leucocytes presence, suggested cardiac muscle injury caused by intense exercise (TG_3_), leading us to believe that 7.5% overload exercise for 8 weeks brings real benefits to the animal but also causes clear injuries and further decreases in animal health.

### Exercise-induced changes in the expression of gene products involved in energy metabolism

An important signal for exercise adaptation consists of greater expression of proteins associated with the oxidative metabolism, such as oxidoreductases, mitochondrial membrane transporters [42]. The mitochondrial internal membrane exerts a fundamental role in oxidative phosphorylation and also in electron transport. Moreover, the translocases move through the mitochondrial membrane and have a logical consequence for an important physiological process known as ATP synthesis [43,44]. In eukaryotic cells, the final stage of nutrients oxidation occurs in mitochondria, with fast oxidation of NADH and FADH_2_ produced in glycolysis, tricarboxilic cycle, ß-oxidation of fatty acids and amino acid oxidation.

In this study we found the presence of a NADH-dehydrogenase in all training groups and in the control (Table 1; Figure 2), which developed a key role in electron transfer in the respiratory chain to Q coenzyme [45]. The expression of that isoform of NADH dehydrogenase was clearly improved from control groups to more intensely trained rats (Figure 2), leading to 1.30 fold at TG_3._ It’s important to cite that NADH dehydrogenase here analyses, maybe an isoform and that this up regulation could not be related to all protein class here evaluated. Additionally, it was only in rat hearts from TG_3_ group that the synthesis of membranes translocases was observed at detectable levels by the techniques applied here. These translocases are commonly associated with ATP’s and H^+^ transport to cytosol (Table 1).

Howlett and Willis (cited by [46]) observed that the isocitrate dehydrogenase (IDH) activity is higher in mitochondria from striated muscle, suggesting that it may rely on this enzyme as a regulatory site. This enzyme catalyzes the first dehydrogenase reaction in the TCA cycle and produces NADH + H^+^, which is the substrate of NADH dehydrogenase. This may explain an over expression of NADH dehydrogenase, as observed in TG_3_ in the present study (Figure 2). Although mitochondria exert the essential role of ATP production, they are also the primary source of cellular reactive oxygen species [47,48]. In a recent review Powers and Jackson [49] suggested that common metabolic changes and ROS generation may predominantly occur by contracting skeletal and heart muscle during different exercise protocols. An exception to this rule is an experiment whereby muscle damage occurs, and in this situation, inflammatory processes may play an important role in radical production. It is well accepted that exercise provides intrinsic protection to the heart [50]. Recent reports have associated ROS production with apoptosis after physical effort, a situation in which the apoptotic mitochondrial pathways may play a major role by releasing cytochrome c and activating initiators such as caspases [46]. An increase in mitochondrial oxidant production is generally accepted as a cause of myocardial cell loss via apoptosis and necrosis [50]. These data corroborates with myocite modifications observed in TG_3_ by microscopy (Additional file 1: Figure S1), explaining the reduction in exercise benefits during the exercise training stage.

During exercise, H^+^ concentration is enhanced, being this process is commonly associated with force generation decline in muscles, also causing a reduction in cross bridge activation by competitively inhibiting Ca^2+^ binding to troponin C. Moreover, proton concentration reduces Ca^2+^ - ATPase re-uptake in sarcoplasm and inhibits myofibrillar ATPase [40]. Mitochondrial ROS generation can lead to a calcium overload, consequently decreased ATP production, and may cause the mitochondrial permeability transition pore (PTP) to open, further decreasing ATP production and releasing cytochrome c. However, the increase of translocases can increase the transport of H^+^ reducing the competition with calcium during the exercise, which would make the most of the positive effect of the exercise.

Finally, another important gene product here detected and probably involved in the increase of ATP synthesis consists in an isoform of magnesium homeostasis factor. homolog, MRS2, 1.5 fold (Figure 2, Table 1), a major Mg^2+^ mitochondria’s transporter being their function extremely important for respiratory complex I and cell viability maintenance [51]. This protein family is characterized for a conserved GMN C-terminus (Gly-Met-Asn) in the transmembrane domains. Furthermore, this is the region responsible for Mg^2+^ selective filter [52] This divalent ion is abundant inside the cell and plays a fundamental role in many biochemical and regulatory functions being his concentration maintained by an transmembrane electrochemical potential [51]. In cardiac muscle Mg^2+^ may be involved in the ATPase phosphate-release step causing inhibition of myofibril sarcoplasmic reticulum Ca^2+^-transporting ATPases under anoxia. This last condition could be improved by high intensity contraction in maximal exercise, when the ATP-PCr system can occur to maintain a relatively constant energy supply [53]. This finding in the present study is in accordance with the increase of aerobic capacity by mitochondrial biogenesis and/or workload improvement as a consequence of swimming training but, one more time, it’s important to cite that maybe an isoform and that this up- or down regulation could not be related to all protein class here evaluated.

## Conclusions

In summary, our study evidenced left ventricular hypertrophy and these data seems to be correlated at molecular levels with proteins of high molecular masses. This increase suggests a clear correlation with the level of intensity which the individuals underwent. It demonstrates that interval training with high intensity compared with low and moderate intensity training led to a remarkable increase in α-MyHC and troponin expressions in the left ventricle of cardiac myocyte of *R. norvegicus*. Another important data is the significant degree of cellular injury in left ventricle in individual submitted to high intensity, when compared with the individuals submitted to low and moderate intensities. Thus, we conclude that 7.5% overload exercise for 8 weeks may possibly improve contractile function for the animal, but may also cause injuries and consequently, reduce the animal’s health. These modifications seem to be related to modifications in contractile and metabolic proteins, previously elucidated by proteomics and molecular analyses. Data here reported add more knowledge to molecular exercise studies.

## Methods

### Animal group design

All procedures are in accordance with the ethics guidelines for research at the University of Brasília and were approved by the ethics committee (UnbDOC n.48695/2010). Twenty isogenic male Wistar adult rats (*Rattus novergicus*), with age varying from 80–90 days, were equally randomized into four groups, one being the sedentary negative control group (CG) and three the swimming-trained groups (TG’s). The control group was maintained in isolated cages receiving water and food *ad libitum*.

### Exercise training protocol

**Figure 3:**
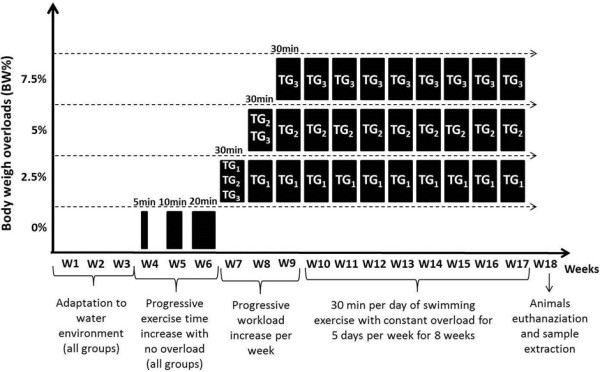
**Exercise training period.** Overview from the exercise experiment design indicating the progress of time and overload trough 8 weeks of swimming exercise.

The animals were adapted to the water environment for three weeks, in the same place as the training sessions, in a cylindrical training tank with a smooth surface, measuring 60 cm in diameter by 120 cm in depth, kept at a constant temperature (32 ± 0.5°C). The training period was corporate to 5 consecutive days of 30 min of swimming sessions for 8 weeks . Training groups were characterized by the overloaded applied, respectively 2.5% (TG_1_), 5.0% (TG_2_) and 7.5% (TG_3_) (Figure 3). The overload was determined weekly by individual animal body weight and attached to the animal’s chest. Aiming to minimize the animal’s stress without promoting physiological adaptations derived from physical exercise, training group animals were submitted to the water environment before the swimming exercise protocol started. At the end of training period, all animals immediately underwent euthanasia after the last training session by decapitation after being anaesthetized with 90 mg.Kg^-1^ of ketamin and 10 mg.Kg^-1^ of xilasin provided by muscle injection.

### Measuring the animals’ training adaptation

In order to verify the animals’ training status, lactate was evaluated and used as parameter for rat adaptation to aerobic performance. At the beginning, (in the first week) and at the end (in the last week) of the training process, maximal lactate steady state (MLSS) was measured by an incremental load swimming test with overload increased by 1% of body weight each 3 min, with 90 s of interval until weariness [54]. Moreover, 25 μL of blood samples were collected from a tail tip cut during the exercise tests and deposited in tubes containing 50 μL of 1% sodium fluoride. MLSS was measured in a lactometer model YSI 2700 S™ (Yellow Springs Inc. – USA). The variable maximum lactate used for MLSS identification was 0.07 mM.min^-1^.

### Heart preparation

Complete heart of each animal was removed and placed in an RNAse free sterile apparatus. The left ventricle was separated in three parts. Two were frozen in liquid nitrogen and stored at −80°C until protein and RNA extractions. The third section was stored in 10% formol for 48 h.

### Histological sample preparation

After formol (10%) fixation, samples were paraffin embedded and sliced in 3 to 4 μM thickness. After the histological sectioning, tissues were stained with hematoxylin and eosin and further analyzed in an Eclipse E200 POL™ optical microscope (Melville, NY).

### Protein extraction

Muscle proteins were extracted from rat heart left ventricles. An amount of 150 mg from each sample was homogenized in 10 mM Tris-EDTA buffer pH 7.4 containing 25 mM sucrose, 2 mM EDTA, and further centrifuged at 1,000 g at 22 to 25°C, as previously described by Short et al*.*[55]. The pellet, containing the myofibrillar fraction, was re-homogenized in 10 mM Tris–HCl buffer pH 7.2 containing 176 mM KCl and 2 mM EDTA. Bradford's method [56], was used for protein quantification. After that, an aliquot was adjusted to a concentration of 3 mg.ml^-1^ and then dissolved fourfold in sample loading buffer (66 mM Tris–HCl pH 6.7 containing 19 mM EDTA, 1.0% v/v SDS, 0.008% v/v bromophenol blue, 810 mM β-mercaptoethanol and 40% v/v glycerol).

### Electrophoretical analyses

SDS-PAGE 12% was performed according to Laemmli [57]. The muscle sample was then loaded on 0.75 mm-thick polyacrylamide gels for protein separation. The discontinuous gel recipe was used, consisting of an 8% separation gel, a 4% stacker gel, and 30% glycerol in the gel matrix. Samples were run in triplicate with CG and TG’s samples in adjacent lanes. Gels were run for 20 h at 275 V. Gels were fixed overnight in 50% vol/vol methanol and 10% vol/vol acetic acid. Gels were silver stained using a protocol suggesting by Blum *et al*. [58] and repeated in triplicate.

Isoelectric focusing and molecular mass separation for 2-DE were conducted according to Gorg et al*.*[59], with minor modifications obtained from Short et al*.*[55] using 13 cm immobilized pH gradient (IPG) strips with a 3–11 pH range and a Multiphor II™ electrophoresis system (GE HealthCare). A total of 500 μg of each extract was precipitated using a 2D Clean-Up Kit (GE HealthCare™) and re-suspended in 250 μl solution of 2% CHAPS, 8 M urea, 7 mg.ml^-1^ dithiothreitol (DTT) and 2% IPG Buffer. Strips were hydrated in this solution for 16 hours. Isoelectric focusing was carried out in a gradient mode for 30 min at 500 V, 90 min at 1000 V, 90 min at 3500 V and 6 hours at 3500 V at 2 mA and 5 W. After the first dimension, strips were equilibrated in a solution of 6 M urea, 1% DTT and 2% SDS for 15 min and then applied to gels. A second dimension for high molecular mass proteins was performed in 18 × 16 × 0.2 cm SDS-PAGE 8% gels [55]. Electrophoresis was conducted in a Hoefer system (GE HealthCare™) at 250 V, 40 mA and 10 W for 8 h. The broad range of isoelectric point marker (GE HealthCare™) was also used for subsequent pI identification on gels.

### In silico gel analysis

All gels were screened on an HP scanner, model Scanjet 8290 and afterwards analyzed by BioNumerics™ v. 4.5 (Applied Maths™) software. To analyze all the gel images, they were all converted to TIFF files. A calibration curve was applied to convert all colors into gray tones (16-bit, 600 dpi) and convert them into pixel values. All technical replicates were aligned and screened by the software in order to identify the same vectors. Every artifacts and unreliable spots were eliminated by manual inspection. An unreliable spot was defined as not appearing on each of the gel images of the same sample. The spots were located and analyzed by their molecular mass and isoelectric point, concentration and matched by their similarity and spot area and densitometry (volume: pixels per inch) were also counted. In order to define the spot’s differential expression a linear regression was performed by the software and a correlation cut off was applied and gels with R^2^ lower than 0.8 were discarded. Mean value of spot volume (dpi) from all three replicate were using to describe the spot density and for further comparison of protein expression, taking into account spots with relative volume equal to or bigger than 0.1 dpi. The spots had to present at least a one fold change were select to comparison with the CG 2D gel with the higher R^2^. Student΄s *t*-test was applied and differences of *p* < 0.05 were considered significant.

### Protein identification by MALDI-TOF

All spots defined by technical reproducibility described above and increase or decrease at least one fold when compared to the same spots in control group (p ≤ 0,005) were excised from gels using a scalpel and each one placed in a 1.5 μl micro tube. Protein in-gel digestion was carried out with Gold sequencing grade trypsin (Promega™) according to Shevchenko et al*.*[60]. 300 μl of 100% acetonitrile was added to tubes for 5 min. Supernatant was removed and spots were dried in a SpeedVac for 5 min. Samples were incubated for 60 min at 56°C in a solution containing 50 μl of 10 mM DTT and 100 mM NH_4_HCO_3_. The solution was replaced with 50 μl of 55 mM iodoacetamide and 100 mM NH_4_HCO_3_ and incubated in darkness for 45 min. Spots were rinsed twice with MilliQ™ water (Millipore™) for 10 min, and exposed to 100 μl of 100% acetonitrile for 5 min. Excess acetonitrile was removed and again spots were dried in a SpeedVac for 5 min. Protein digestion was carried out using 650 ng trypsin diluted in 50 μl of 50 mM NH_4_HCO_3_ and 6 mM CaCl_2_, with overnight incubation at 37°C. The peptides derived from tryptic digestion were analyzed as described by Henzel et al*.*[61] using an UltraFlex II™ MALDI-TOF (Matrix-Assisted Laser Desorption Ionization Time-of-Flight, Bruker Daltonics™, Billerica, MA). A sample of 2 μl was mixed in 6 μl of 0.1% α-cyano-4-hydroxycinnamic acid, 0.1% trifluoroacetic acid dissolved in acetonitrile (1:1). A volume of 0.5 μl was applied to a MALDI plate and dried at room temperature. Spectrometry was operated in a linear mode for MS acquisition and reflected mode for MS/MS acquisitions using modulated power with 200 random shoots. Data were saved in standard Bruker software format. Spots were identified using Peptide Mass Fingerprinting (PMF). Peptide mass lists were produced automated analysis tools of mass spectrometer previously described [62]. Data were smoothed (Gaussian, 2 chan peak width), baseline subtracted (100 chan peak width) and an adaptive (× 8.0) threshold applied as described by Holloway *et al*. [63]. Monoisotopic peak (25% centroid) selection was restricted to 15 peptides over 600–40000 m/z, and the peak list searched against the Swiss-Prot database restricted to *Rattus novergicus* databank using a locally implemented MASCOT ( http://www.matrixscience.com) server. The enzyme specificity was trypsin, allowing one missed cleavage, carbamidomethyl modification of cysteine (fixed), oxidation of methionine (variable). Protein identification was accepted based on a significant Mowse score

### Quantification of MyHC mRNA by real time qPCR

Total RNA was extracted with the Trizol™ reagent (Invitrogen™, USA) from 25 mg of each animal’s ventricle using standard protocol. Total RNA from individual animals from different training groups was pooled together. This provided RNA samples from the untrained group and from the other groups: 2.5% (TG_1_)_,_ 5.0% (TG_2_) and 7.5% (TG_3_). The cDNA was generated using a High Capacity cDNA Archive Kit (Applied Biosystems™) following the manufacturer’s instructions. A TaqMan Gene expression assay for myosin, heavy polypeptide 6, cardiac muscle, alpha (*Myh*6, Rn00568304_m1) was bought from Applied Biosystems™ (Foster City, CA). Equal amounts of RNA (0.5 μg) were reverse transcribed using High Capacity cDNA Archive (Applied Biosystems™) and submitted to qPCR. TaqMan assays were carried out with a StepOnePlus instrument (Applied Biosystems™, Foster City, CA) in 20 μl reactions containing 0.5 μl of TaqMan Gene expression Assays (20 ×), 12,5 μl of TaqMan Universal PCR Master Mix (2 ×) and 2 μl of template cDNA (100 ng). After initial denaturation at 95°C for 10 min, amplifications were carried out in 40 cycles at 95°C/15 s and 60°C/1 min. Comparative CT (crossing threshold) method was used to establish differential expression among training groups and control. The constitutive *rRNA18S* gene expression was used for data normalization. Relative quantitation was carried out using ΔΔC_T_ method based on three technical replicates.

## Abbreviations

α-MyHC: α -myosin heavy chain; β- MyHC: β-myosin heavy chain; 2-DE: two-dimensional electrophoresis; ATP: adenosine triphosphate; cDNA: complementary deoxyribonucleic acid; CG: control groups; CHAPS: 3-[(3-cholamidopropyl)dimethylammonio]-1-propanesulfonate; cTn: cardiac troponin; DTT: dithiothreitol; EDTA: ethylenediaminetetraacetic acid; FADH_2_: flavin adenine dinucleotide, hydroquinone form; IEF: isoelectric focusing; IPG: immobilized pH gradient; MALDI-ToF: matrix-assisted laser desorption ionization time-of-flight; MLC: myosin light chain; MLSS: maximal lactate steady state; Mn-SOD: manganese-superoxide dismutase; mRNA: messenger ribonucleic acid; MS: mass spectrometry; MS/MS: tandem mass spectrometry; MyHC: myosin heavy chain; NADH: nicotinamide adenine dinucleotide at reduced form; NCBI: National Center for Biotechnology Information; PMF: peptide mass fingerprinting; qPCR: real-time polymerase chain reaction; R^2^: coefficient of linear regression determination; RNA: ribonucleic acid; SDS-PAGE: sodium dodecyl sulphate polyacrilamide gel electrophoresis; TCA cycle: tricarboxylic acid cycle; TG_1_: TG_2_ and TG_3_, training groups with overload of 2.5%, 5.0% and 7.5% respectively; Tris-EDTA: hydroxymethyl aminomethane-ethylenediaminetetraacetic acid; Tris–HCl: ethylenediaminetetraacetic acid - Hydrochloric acid.

## Competing interests

Authors declare there are absolute not political, personal, religious, ideological, academic, intellectual, commercial conflict of interests in this work.

## Authors’ contributions

LAOR it conceived and participates of design of the study, exercise training, electrophoretical and *in silico* gel analysis and drafted the manuscript. BP participates of exercise training and electrophoretical analyses. DHB does the Histological sample preparation and analyses. RVO helped to draft the manuscript. RV participates of molecular studies (real time qPCR). GD carried out the protein identification by MALDI-TOF. RWP participates of molecular studies (real time qPCR) and helped to draft the manuscript. OLF participates conception and design and coordination of the study and helped to draft the manuscript. All authors read and approved the final manuscript.

## Supplementary Material

Additional file 1 **Figure S1.** Cross-sections of left ventricle. Crosssections of left ventricle from rats heart stained with haematoxylin and eosin for hystopathological analysis. A) Cardiac fibers from left rats ventricles pertaining to CG with a normal size and shape. B) TG1 with a normal aspect indicating no clear pathological alteration. C) TG2 shows a small sclerotic area (I) with infiltration of granule lipofuscin (I). D) TG3 shows areas with increased fibrosis (II) and an extended area with necrosis (III). N marks the nucleus in groups A, B and C. The image was magnified by 400 times and photographed with a camera Sony™ model DSC-H1 (MyHC). Click here for file

Additional file 2 **Figure S2.** SDS-PAGE MyHC isoforms. SDS-PAGE silver stained control group (CG). TG1; TG2 and TG3 correspond respectively to 2.5; 5.0 and 7.5 to overload training groups. The arrow indicates the myosin heavy chain. Click here for file

Additional file 3 ** Figure S3. **Two-dimensional electrophoretic evaluation of left ventricle high molecular mass proteins. Representative silver stained high-molecular mass two-dimensional gel (A) and further spot density analyses (B). The arrows indicate the selected spots further identified by MS analysis. Click here for file
