# Supplementary figures and images for: High molecular mass proteomics analyses of left ventricle from rats subjected to differential swimming training
Source: BMC Physiol. 2012 Sep 5;12:11. doi: 10.1186/1472-6793-12-11 (PMC3508799; doi:10.1186/1472-6793-12-11)

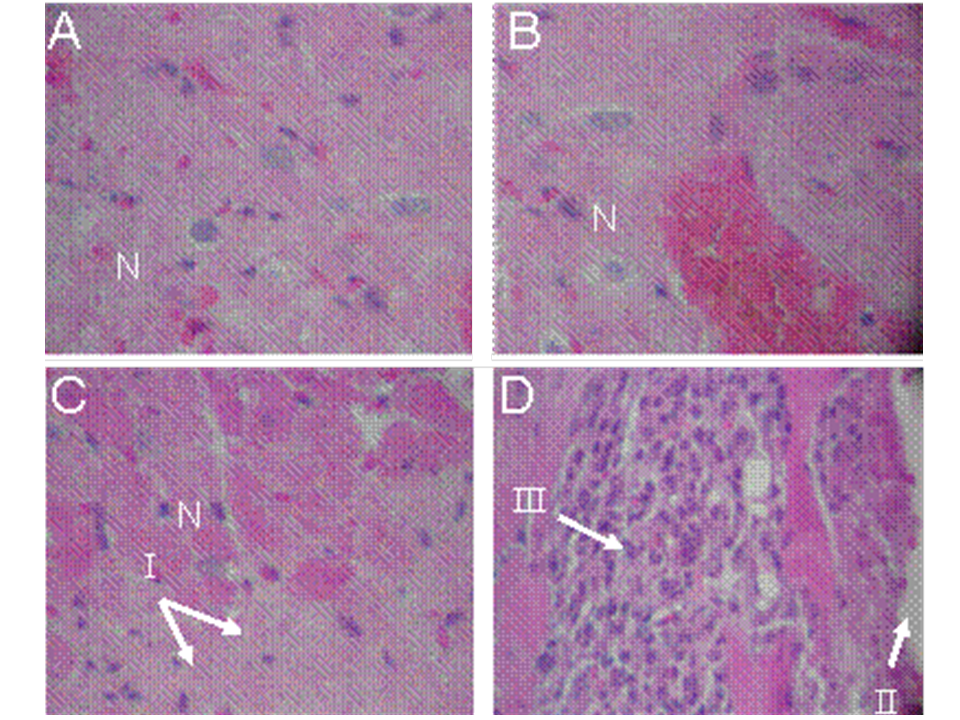

Supplement: Additional file 1 — Figure S1. Cross-sections of left ventricle. Crosssections of left ventricle from rats heart stained with haematoxylin and eosin for hystopathological analysis. A) Cardiac fibers from left rats ventricles pertaining to CG with a normal size and shape. B) TG1 with a normal aspect indicating no clear pathological alteration. C) TG2 shows a small sclerotic area (I) with infiltration of granule lipofuscin (I). D) TG3 shows areas with increased fibrosis (II) and an extended area with necrosis (III). N marks the nucleus in groups A, B and C. The image was magnified by 400 times and photographed with a camera Sony™ model DSC-H1 (MyHC). [file 1472-6793-12-11-S1.tiff]

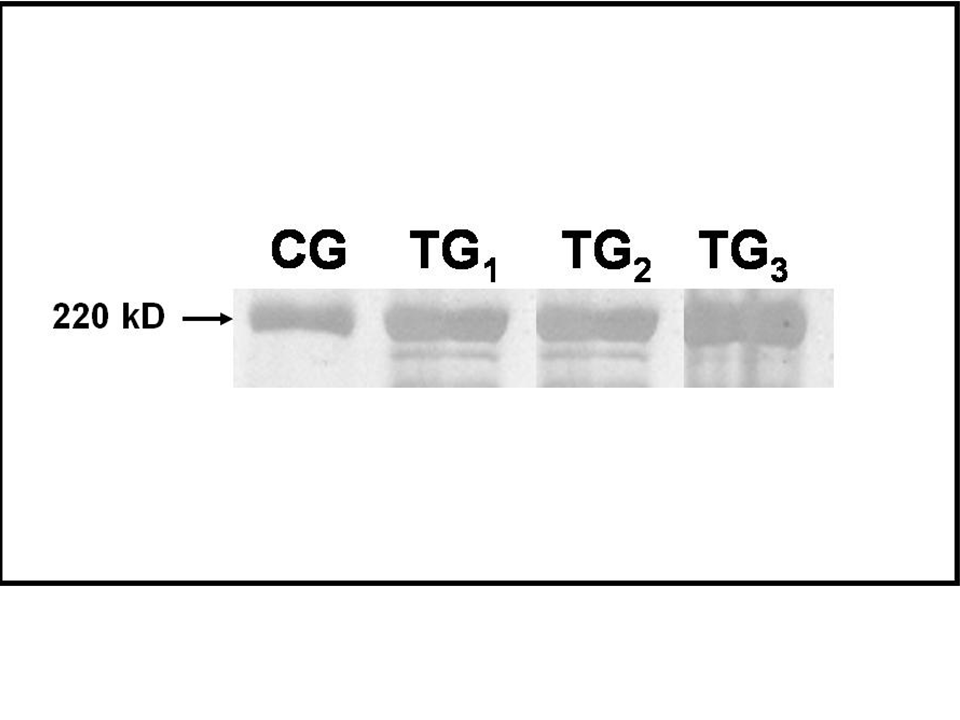

Supplement: Additional file 2 — Figure S2. SDS-PAGE MyHC isoforms. SDS-PAGE silver stained control group (CG). TG1; TG2 and TG3 correspond respectively to 2.5; 5.0 and 7.5 to overload training groups. The arrow indicates the myosin heavy chain. [file 1472-6793-12-11-S2.tiff]

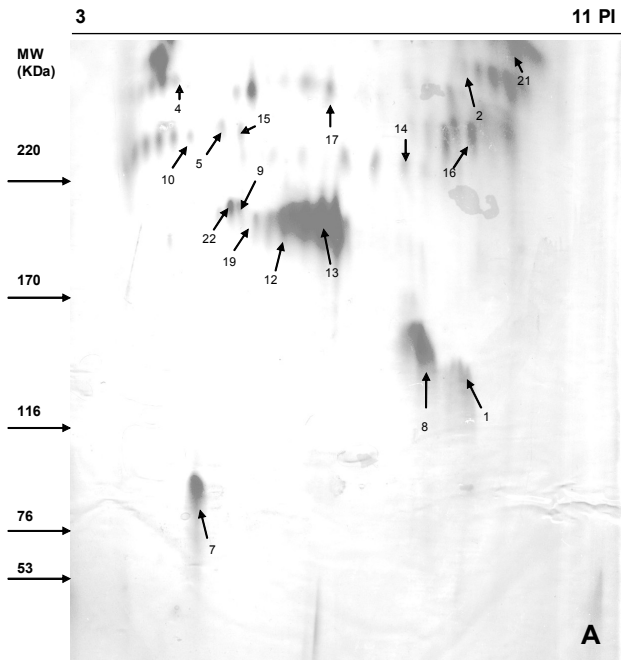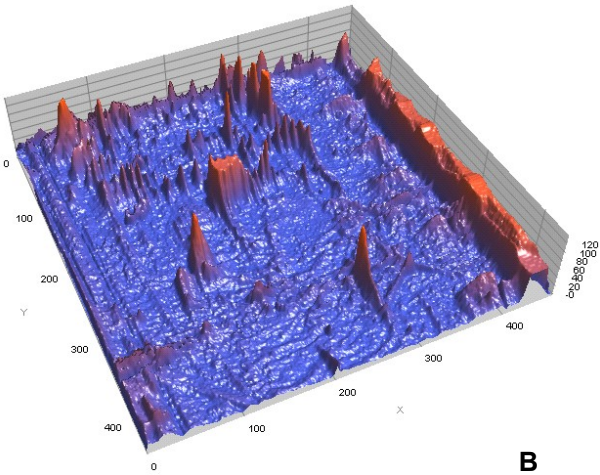

Supplement: Additional file 3 — Figure S3. Two-dimensional electrophoretic evaluation of left ventricle high molecular mass proteins. Representative silver stained high-molecular mass two-dimensional gel (A) and further spot density analyses (B). The arrows indicate the selected spots further identified by MS analysis. [file 1472-6793-12-11-S3.pdf]
